# Impact of Red Blood Cell Deformability and Local Hematocrit on Microvascular Flow Resistance: Insights from In Vitro Microfluidic Measurements and Viscosity Modeling

**DOI:** 10.3390/life16081382

**Published:** 2026-08-21

**Authors:** Efstathios Kaliviotis, Pavlos Stephanou, Stavroula Balabani

**Affiliations:** 1 Department of Mechanical Engineering and Materials Science and Engineering, Cyprus University of Technology, Limassol 3036, Cyprus; 2 Department of Chemical Engineering, Cyprus University of Technology, Limassol 3036, Cyprus; pavlos.stephanou@cut.ac.cy; 3 Department of Mechanical Engineering, University College London, Torrington Place, London WC1E 7JE, UK; s.balabani@ucl.ac.uk

**Keywords:** Y-type microchannel blood flow, blood rheology modelling, RBC concentration and deformability

## Abstract

Altered red blood cell (RBC) deformability can impact microvascular flow resistance through changes in cell partitioning at bifurcations, and local hematocrit distribution, a key determinant of the effective viscosity of blood. In this work we report on resistance-relevant viscosity distributions extracted from in vitro microfluidic observations of a Y-junction type blood flow. Spatially resolved hematocrit distributions in the parent channel and daughter branches, determined by experimental measurements in earlier work, for healthy and stiffened RBC suspensions over a range of daughter to parent flow-split ratios, $Q^{*}$, are combined with a recently proposed viscosity model enabling the estimation of the local relative viscosity profiles $\eta_{r}$. The maximum of the RBC elongation index EI, was utilized to define of the deformability parameter λ, which with the local RBC volume fraction, Φ(y), were used as inputs in the relative viscosity, $\eta_{r}$. The latter is a modified Krieger–Dougherty viscosity expression, accounting explicitly for deformability and RBC concentration. The resulting viscosity profiles are averaged in the channel regions of interest to provide an apparent viscosity in the branches, used here as an indicator of microvascular flow resistance. The results illustrate that deformability and hematocrit-dependent RBC enrichment or depletion in the branches of a vascular junction may amplify or attenuate the resistance asymmetry in the daughters of the Y-junction.

## 1. Introduction

Microvascular blood flow is governed by the complex interplay between red blood cell (RBC) mechanics, local hematocrit distribution, and vascular geometry. In small vessels and microfluidic systems, blood cannot be treated as a homogeneous fluid; instead, it behaves as a suspension of aggregating deformable particles, where spatial variations in RBC concentration significantly influence the local blood rheology and consequently the apparent viscosity and blood flow resistance [1,2,3]. A key determinant of microvascular flow is the ability of RBCs to deform under shear. RBC deformability allows the passage of the cells through narrow capillaries and enhances phenomena such as lateral migration, which contributes to the formation of cell-depleted layers near vessel walls and results in heterogeneous concentration profiles [4,5,6]. Alterations in deformability, such as those observed in pathological conditions including diabetes, malaria, and sickle cell disease, are known to impair microcirculatory perfusion and increase flow resistance [7,8,9].

At vascular bifurcations, the distribution of RBCs from the parent to daughter branches is governed by the well-established phase separation (plasma skimming) phenomenon, whereby RBCs preferentially enter branches with higher flow rates [10,11,12]. This results in non-uniform hematocrit partitioning, which has been shown to strongly affect local viscosity and vascular resistance [1,2,13]. Experimental and theoretical studies have demonstrated that even small asymmetries in flow partitioning can lead to significant differences in hematocrit between branches, thereby altering downstream hemodynamics [14,15]. In addition to concentration effects, RBC deformability influences suspension rheology through both direct and indirect mechanisms. Directly, deformability affects the intrinsic viscosity of the suspension, as deformable particles exhibit different stress responses compared to rigid particles [16,17]. Indirectly, deformability alters particle migration, distribution, and shear-induced diffusion, thereby modifying local hematocrit fields and consequently the spatial distribution of viscosity [6,18,19,20].

Recent advances in microfluidics have enabled detailed measurements of RBC concentration and deformability in controlled geometries, such as T- and Y-type bifurcating channels [6,12,13,20]. However, translating these spatially resolved measurements into effective resistance metrics remains a challenge. In particular, the relationship between local RBC concentration and local viscosity should be appropriately defined in order to determine the apparent resistance in the region. This relationship is non-trivial because apart from the complex nature of blood rheology [21,22], the viscosity, hematocrit, and velocity are spatially coupled; thus different concentration distributions can produce substantially different local hemodynamic responses even when the bulk hematocrit is similar [13,23,24,25].

In this work, we address this issue by combining experimentally derived hematocrit distributions from Stathoulopoulos et al. [20] with a deformability-dependent viscosity model based on the approach of Polykarpou et al. [26]. The objective is to estimate both local and effective viscosity in a Y-type junction microchannel and to discuss how RBC deformability and flow partitioning may influence microvascular resistance. By linking microfluidic observations to resistance-relevant viscosity quantities, this study aims to provide a framework for interpreting microvascular flow behavior in both physiological and pathological conditions.

## 2. Materials and Methods

### 2.1. Experimental Data

**Figure 1:**
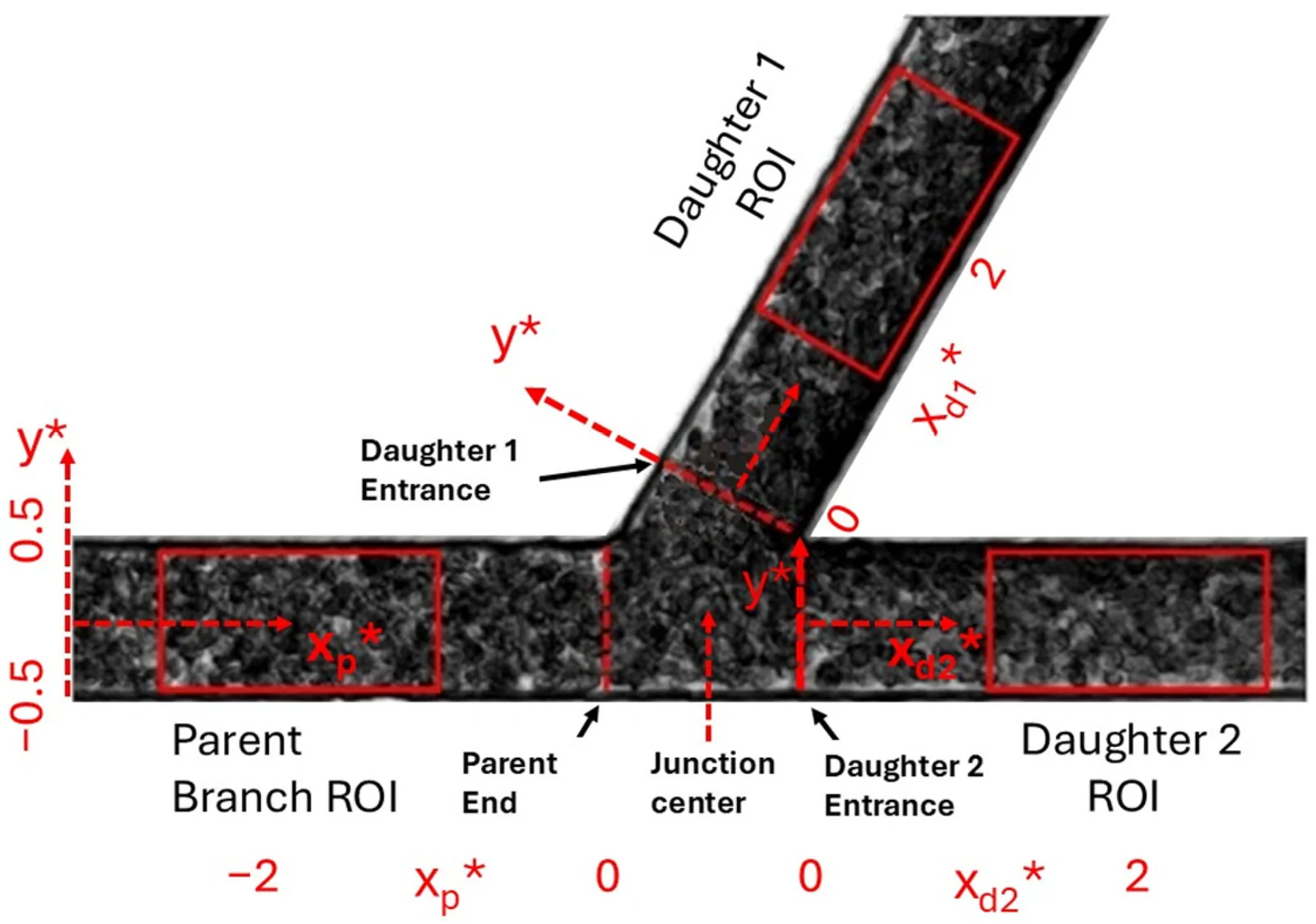
Diagram of the microchannel with geometry details (reproduced from Stathoulopoulos et al. [20]). y* and x* denote the cross-flow and axial channel dimensions respectively, normalised with the channel width of 50 μm.

Experimental data are utilized from the work of Stathoulopoulos et al. [20]. In this work, the blood flows of normal and hardened RBC solutions were perfused in a Y-type asymmetric channel (Figure 1). The microchannel had two daughter branches: one directly opposite the parent branch (noted here as Daughter 2), and another one (Daughter 1) at 45° angle with respect to parent and Daughter 2 branches. The channel had a square cross section, 50 × 50 μm width and height (W × H). To facilitate the analysis, several regions and lines of interest were defined. Three regions of interest (ROIs) were defined in the parent and daughter branches respectively, with a length of two channel widths (2W), to provide statistically meaningful spatially averaged hematocrit profiles. The ROIs were located at two widths distance from the parent-end and daughter entrances respectively, where the flow and structural profiles are expected to be more uniform compared to those in the Y-junction. Three lines of interest across the channel, located at the parent-end and at the entrances of the daughter branches, were also defined as shown in Figure 1, to capture the RBC behavior in the bifurcation region.

The blood samples in [20] were collected from human volunteers (anticoagulated with EDTA at a concentration of 1.8 mg mL^−1^) in accordance with the guidelines and regulations of the South East London NHS Research Ethics Committee (Reference: 10/H0804/21). RBC suspensions were prepared by separating RBCs from whole blood via centrifugation, followed by two washing steps in phosphate-buffered saline (PBS). The final hematocrit (Hct) was adjusted to 20% in PBS for the microfluidic scales employed in this study. RBC rigidification was achieved through treatment with a 0.08% glutaraldehyde (GA) solution for 15 min, following established protocols. All samples were obtained from a single donor to minimize inter-donor variability in RBC properties.

### 2.2. RBC Concentration and Deformability

RBC deformability was quantified using a microfluidic ektacytometer (Rheoscan-D300, RheoMeditech, Seoul, Republic of Korea). Measurements were performed in triplicate for both whole blood and RBC suspensions, and the mean maximum elongation index (EI_max_) was calculated, from the EI/stress response obtained from the D300 instrument. The EI is obtained from the major (L) and minor (W) axis of an ellipsoidal diffraction pattern, produced when cells undergo deformation in the ektacytometer: $EI=\frac{L-W}{L+W}$. The EI_max_ values were 0.54 for whole blood, consistent with previously reported values for normal cells [27]. GA-treated RBCs exhibited a reduced EI_max_ of 0.30, corresponding to an approximate 40% decrease in deformability compared to untreated cells.

The local RBC concentration $\Phi \left(y^{*}\right)$ was obtained from the normalized hematocrit profiles $Hct^{*}(y^{*})$ reported in Stathoulopoulos et al. [20], by directly scaling the data with the feed hematocrit of 20% employed in that work, which was effectively the normalizing value used therein. The hematocrit profiles represent median values across a range of flow-split ratios investigated in that work, in the daughter branches (see next section). The local RBC concentrations were spatially averaged (*Φ*_av_) within specific regions of interest using the arithmetic mean of $\Phi (y^{*})$.

### 2.3. Flow Rate Ratios

The flow rate ratio Q* is defined as the ratio of the flow rate in the daughter branches (Qd_1_, Qd_2_) to the flow rate in the parent branch Q_p_:

(1) Q* = Qd_1,2_/Q_p_

The flow rates in the daughter branches were adjusted so that the side branching Daughter 1 received the lower flow rates (0 < Q* < 0.5) and the direct outlet branching (Daughter 2) received the higher flow rates (0.5 < Q* < 1). Therefore, the hematocrit measured in Stathoulopoulos et al. [20], for the two different Q* ranges tested in the daughter branches, should reflect an indicative RBC concentration behavior resulting from either low (Q*_low_ in Daughter 1) or higher (Q*_high_ in Daughter 2) flow rate ratios.

### 2.4. The Viscosity Model

The viscosity model is based on the work of Polykarpou et al. [26], adapting the diffusive flux constitutive model of Phillips et al. [28], under steady-state conditions, for large Peclet and low Reynolds numbers. A brief description of the model is provided below:

∇⋅u=0

(2) −∇p+∇⋅σ=0

$$\nabla \cdot \left(N_{c}+N_{\eta }\right)=0\;N_{c}=-K_{c}a^{2}\Phi \nabla \left(\Phi \dot{\gamma }\right)\;N_{\eta }=-K_{\eta }a^{2}\frac{\Phi^{2}\dot{\gamma }}{\eta \left(\Phi \right)}\nabla \eta (\Phi )$$

In the above continuity, momentum and concentration evolution equations, **u** is the velocity field, **σ** is the stress tensor, *p* is the thermodynamic pressure field, $\dot{\gamma }$ is the local shear rate, ∇ is the nabla operator, and a is the radius of the particles. $\Phi (y^{*})$ is the local dimensionless particle/cell concentration, the evolution of which affects (a) the spatially varying particle collision frequency $N_{c}$, via the diffusion coefficient $K_{c}$, and (b) the spatially varying viscosity $N_{\eta }$ through the diffusion coefficient $K_{\eta }$ and the concentration dependent viscosity $\eta \left(\Phi \right)$. In steady state, the phenomena occurring due to $\Phi (y^{*})$ are balanced through the relationships in Equation (2), and only the ratio of the coefficients $K_{c}$ and $K_{\eta }$ needs to be specified. For radially dependent flows, the equation for $\Phi (y^{*})$ is reduced to [26,28].

(3) $$\frac{\dot{\gamma }}{{\dot{\gamma }}_{w}}\;\frac{\Phi }{\Phi_{w}}={\left(\frac{\eta \left(\Phi_{w}\right)}{\eta \left(\Phi \right)}\right)}^{\beta },$$

where $\beta \equiv K_{\eta }/K_{c}$, and $\Phi_{w}$ and ${\dot{\gamma }}_{w}$ are wall values of the cell concentration and shear rates respectively. The shear stress σ, developed in a Newtonian solvent with viscosity $\eta_{s}$ due to the particle concentration, was described using the generalized Newtonian model by Phillips et al. [28] as:

(4) $$\sigma =\eta \left(\Phi \right)\dot{\gamma }=\eta_{p}\eta_{r}\left(\Phi \right)\;\dot{\gamma },$$

where $\eta_{r}(\Phi )\equiv \eta /\eta_{p}$ is a Krieger-stated relative viscosity of the form [29]:

(5) $$\eta_{r}\left(\Phi \right)={\left(1-\frac{\Phi }{\Phi_{m}}\right)}^{-b}$$

$\Phi_{m}$ is the maximum volume (packing) fraction, with values reported in experimental and theoretical studies between 0.62 and 0.70 for the case of hard RBCs [30,31]. In the present case, values of 0.70 and 0.95 were used for the stiffer and normal $\Phi_{m}$ samples respectively [30,31,32]. $\eta_{p}$ in Equation (4) is the solvent viscosity, representing the plasma viscosity of blood.

To incorporate the effect of deformable particles, Stephanou [33] and Stephanou and Tsimouri [34] have proposed utilizing a version of Equation (5) with the exponent *b* modified as follows:

(6) $$\eta_{r}^{*}\left(\Phi ,\lambda \right)={\left(1-\frac{\Phi }{\Phi_{m}}\right)}^{-T(\lambda )\Phi_{m}},\;\;T(\lambda )=\frac{5\lambda +2}{2\left(\lambda +1\right)}$$

$\eta_{r}*$ is the modified Equation (5), $\lambda =\eta_{c}/\eta_{p}$, is the cytoplasmic to plasma viscosity ratio, a key parameter for RBC deformability. Earlier experimental and theoretical studies [35,36] have shown that increasing viscosity of the cytoplasm (the hemoglobin-rich intracellular fluid enclosed by the RBC membrane) reduces the extent of deformation and suppresses the tank-treading motion of the RBC. More recent studies [37,38] confirm a nonlinear decrease in deformation with increasing viscosity ratio, with significant reductions observed for λ > 8. It should be noted, however, that in a recent study on the viscosity and density on RBC cytosol (i.e., the RBC cytoplasm) by John et al. [39], a mean value of $\bar{\lambda }$ = 10 was found, from a log-normal distribution of the cytosol viscosity, due to the variation in RBC age in the cell population of the sample.

In the present study, λ values were set to 5 for the normal RBC cases, a commonly employed value in the literature, and 20 for the stiffer RBCs of the experimental case. A value of λ = 10 was also utilized in the model to investigate the effect of the finding in John et al. [39]. It should be noted that there is very limited information in the literature about the direct relation of the viscosity ratio λ and the deformability index EI_max_. Frank et al. [40] have shown that in sickle cell disease patients with moderate to severe hemoglobinopathy, the EI_max_ index is reduced by approximately 40%, while Byun et al. [41] have shown that the cytoplasmic viscosity in the same pathological condition can reach 23.8 ± 9.5 mPa s. Therefore, a λ value of 20 was considered a reasonable assumption to reproduce an approximately 40% reduction in RBC deformability in the present study.

### 2.5. Apparent Viscosity Estimation—Vascular Resistance Indicator

The apparent viscosity in the regions of interest of the channel (see ROIs in Figure 1) is considered in this work as a vascular resistance indicator of a vessel with similar geometrical characteristics. It should be noted that even though microvascular vessels are often idealized as cylindrical conduits with circular cross-sections, extensive experimental evidence indicates that their actual geometry deviates significantly from this simplification. In vivo imaging studies have demonstrated that microvessels and venules exhibit irregular, non-circular, elliptical, polygonal, or even collapsed geometries [42].

The apparent viscosity in the channel regions of interest was estimated according to previous approaches for flow balanced viscosity, in a circular geometry for simplicity [43,44]. More specifically, the flow rate in a region where the viscosity varies across the channel should be equal to a flow rate for a fluid of constant apparent viscosity flowing in the same region. Τhe equations of motion in cylindrical coordinates, for steady, incompressible, and fully developed flow, with a constant pressure gradient, results in [45]:

(7) $$0=-\frac{dP}{dz}+\frac{1}{r}\frac{d}{dr}\left(r\sigma \left(r\right)\right)\Rightarrow \sigma \left(r\right)=-\frac{r}{2}\left(-\frac{dP}{dz}\right)+C$$

Here $\sigma \left(0\right)=0\Rightarrow C=0$. Then the flow rate reads:

(8) $$Q=2\pi \int_{0}^{1}u_{z}\left(r\right)rdr=-\pi \int_{0}^{1}\left(\frac{du_{z}\left(r\right)}{dr}\right)r^{2}dr,$$

where in the second equation we employed an integration by parts [44]. Since $\sigma \left(r\right)=\eta \left(r\right)\left(\frac{du_{z}\left(r\right)}{dr}\right)$,

(9) $$Q=-\pi \int_{0}^{1}r^{2}\frac{\sigma \left(r\right)}{\eta \left(r\right)}dr.$$

Using $\sigma \left(r\right)=-\frac{r}{2}\left(-\frac{dP}{dz}\right)$ the flow rate for a radially dependent viscosity becomes:

(10) $$Q=\frac{\pi }{2}\left(-\frac{dP}{dz}\right)\int_{0}^{1}\frac{r^{3}}{\eta \left(r\right)}dr,$$

Also, the flow rate for the constant apparent viscosity holds as:

(11) $$Q=\frac{\pi R^{4}}{8\eta_{app}}\left(-\frac{dP}{dz}\right)$$

Thus, by equating the flow rates from (9) and (10):

(12) $$\eta_{app}=\frac{R^{4}}{4}{\left[\int_{0}^{R}\frac{r^{3}}{\eta \left(r\right)}dr\right]}^{-1}$$

In Equation (12), $\eta \left(r\right)$ is the concentration and deformability dependent viscosity of Equation (6), $\eta_{r}\left(\Phi ,\lambda \right)$. For the analysis of the present case, the above expression for the radially (r) dependent viscosity is modified for the channel dimensions of Figure 1, with a half width Y*:

(13) $$\eta_{app}=\frac{{Y^{*}}^{4}}{\;4}\;{\left[\int_{0}^{Y*}\;\;\frac{{y^{*}}^{3}}{\eta_{\left(y*\right)}}dy^{*}\right]}^{-1}\;$$

Equation (13) is solved numerically for both sides of the y* range with a normalized width of Y* = 0.5 (i.e., −0.5 ≤ y* ≤ 0, and 0 ≤ y* ≤ 0.5) to account for asymmetries in $\eta_{\left(y*\right)}$ profile, and $\eta_{app}$ is estimated as the average of the two contributions in the whole channel. The result is corrected for a reduction of 30% in hydraulic resistance in the square, compared to the circular vessel due to the square geometry used here [46].

### 2.6. Statistical Analysis

The Hct* data in the work of Stathoulopoulos et al. [20] were presented as median profiles from the Q* ranges tested in the channel. The distribution of the data across the median values of the profiles was also reported in [20], indicating normality for large portions of y*. Thus, the median profiles indicate to a large degree the most probable behavior of *Φ* in the channel, and therefore the calculation of the average concentration in a channel location as the mean value of *Φ*(y*) was used in the present work.

## 3. Results

### 3.1. Local RBC Concentration Φ(y*)

**Figure 2:**
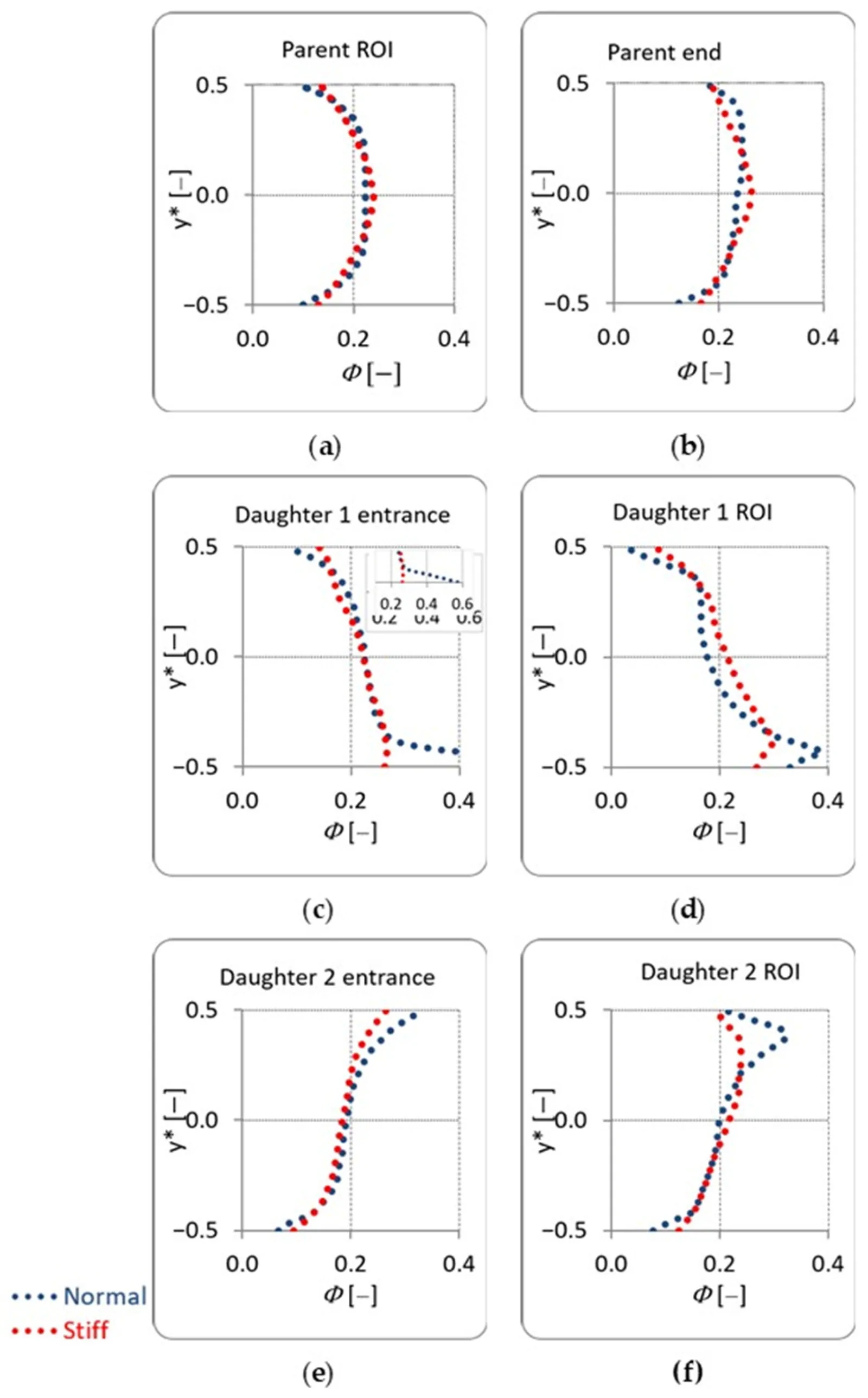
Local distribution of RBC concentration from all flow ratios Q* tested in the parent and daughter branches of the channel. The parent ROI and exit are shown in panels (**a**,**b**). Daughter 1 entrance and ROI are shown in (**c**,**d**). Entrance and ROI of Daughter 2 are shown in (**e**,**f**). The y* axis is aligned with the geometry of the channel as seen in Figure 1.

The RBC concentration *Φ*(y*) was estimated as volume fraction from the normalized hematocrit profiles, Hct*(y*), reported in [20]. The hematocrit profiles in [20] were obtained for different flow ratio ranges in each branch: in Daughter 1, the flow ratio ranged between 0 and 0.5 (0 < Q*_d__1_ < 0.5), whereas in Daughter 2, the flow rate ratio range was from 0.5 to 1 (0.5 < Q*_d__2_ < 1). Figure 2 shows that the concentration *Φ*(y*) scales directly with Hct*(y*) in [20] as explained earlier.

In the Parent ROI, the profiles are both symmetric across the channel central axis; however, the profiles of the stiffer RBC samples appear slightly sharper than those of the normal ones, especially in the Parent-end area. Both the sharpness and symmetry in the profile of the stiff samples persist at the Parent-end region. However, the profile of the normal RBC sample seems to become asymmetric therein, while that of the stiffer cells shows a lesser degree of asymmetry. The main reason for this asymmetry seems to be the difference in the flow rate ratio range between the daughter branches (low vs. high for Daughter 1 and Daughter 2 respectively).

In the entrances and ROIs of Daughters 1 and 2, the symmetry of both sample types has been significantly affected, with the normal RBC samples showing distinctive peaks near the inner walls (i.e., the walls forming the apex of the junction) of the channel. The peak appears larger at the entrance of Daughter 1, which is the daughter of the low flow rate ratio range, reaching magnitudes of 0.6 at the wall. At the ROIs of both daughters the concentration asymmetry persists, however, the peaks of the concentration appear distanced from the inner walls.

**Figure 3:**
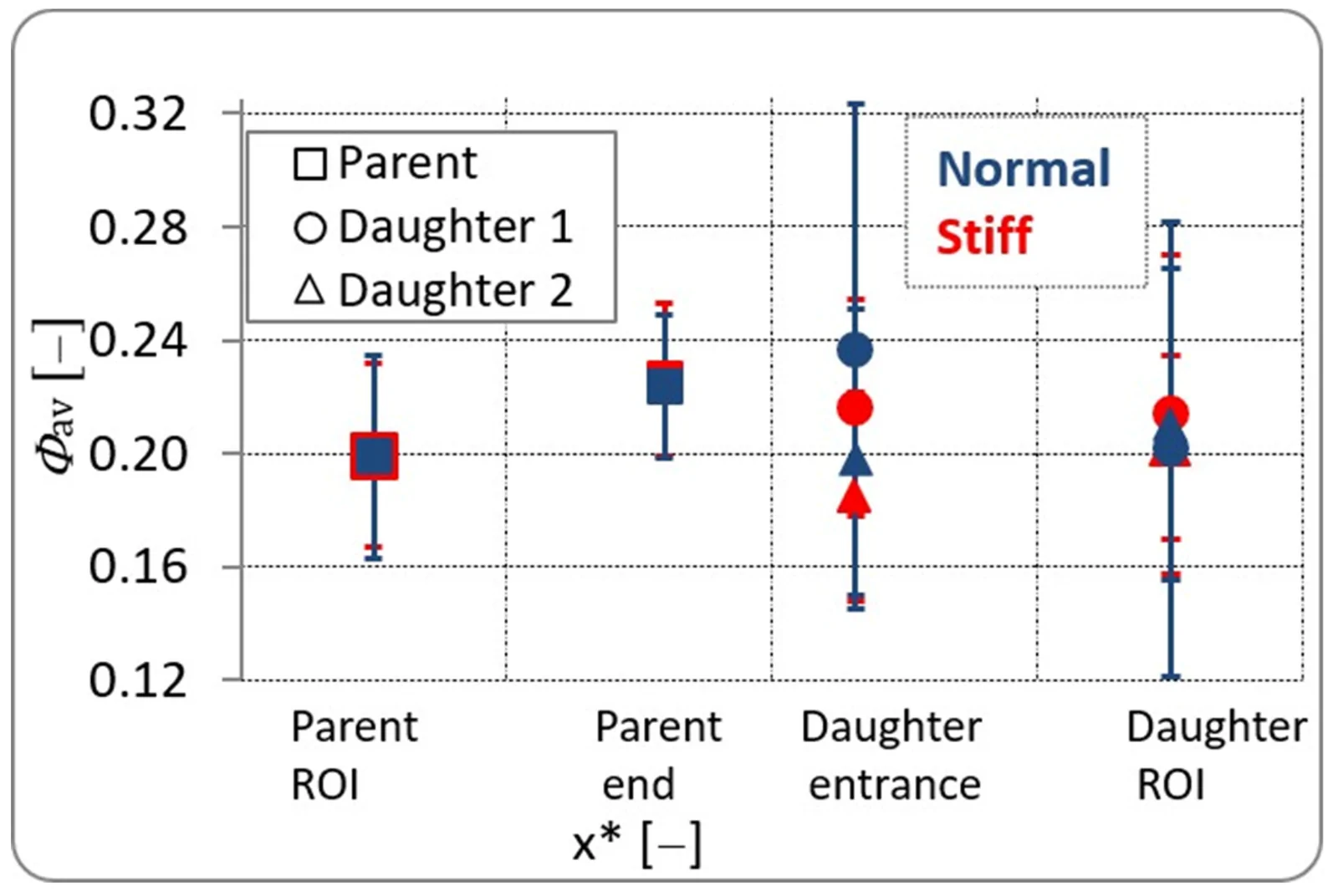
The average RBC concentration in the various locations of the channel.

Figure 3 shows the average RBC concentration, *Φ*_av_, in the various locations and ROIs in the channel, calculated from the concentration profiles, *Φ*(y*), of Figure 2. In the Parent ROI the mean hematocrit matches the feed hematocrit at 20%, as expected. As the flow approaches the region of the Y-junction, the mean RBC concentration increases to similar levels for both normal and hardened suspensions, indicating cell accumulation due to the presence of the bifurcation apex ahead. At the entrances to the daughter branches, *Φ*_av_ is strongly influenced by both the flow rate ratio and cell deformability. At the lower-flow rate daughter (Daughter 1, 0 < Q*_d__1_ < 0.5) a higher concentration is observed compared to Daughter 2 (the higher-flow rate daughter, where $Q^{*}>0.5$) for both samples. This reflects RBC accumulation in the area, associated with changes in the flow direction and flow intensity (velocity and shear conditions). This cell accumulation is more pronounced for the normal RBCs, which consistently exhibit higher local concentrations than hardened cells at the daughter entrances. At the entrance of Daughter 2, which is the outlet of the higher flow rate ratio range, *Φ*_av_ levels of the stiff cells drop below the feed hematocrit seen in the Parent ROI, while the level of the normal cells seems mostly unaffected. This reduction in *Φ*_av_ might be expected, as in the specific branch, the separating velocity streamlines, originating in the parent branch, force a higher flux and faster cell clearance in the area [6]. Furthermore, it is apparent in Figure 3 that the concentration of the stiffer cells is slightly lower than the normal ones in the daughter entrances, suggesting a faster clearance of the stiffer cells in the area since the fluxes of both the normal and stiff cells in [20] are found to be very similar. This is in line with the finding in [6], where rigidified RBCs are found to move faster than the normal ones in similar geometries (daughters of a T-junction) and flow ratios, although in smaller concentrations (up to 5%).

Further downstream, within the daughter ROIs, the mean concentration for all cases converges towards similar values, although it remains slightly higher than the *Φ*_av_ in the parent branch. This can be attributed to the fact that RBC concentration cannot fully recover within such a short distance from the bifurcation; local RBC flux alterations might require a longer development length following the flow split at the bifurcation. The mean hematocrit of the stiff cells in the ROI of Daughter 1 appears slightly higher than the one of normal RBCs, indicating faster passage clearance of the more deformable cell suspension. In Daughter 2 the opposite is observed, although the differences are very small.

Overall, the average hematocrit in the Daughter ROIs does not fully recover to the Parent-branch value, which is consistent with the persistent RBC accumulation in the junction area and the limited downstream distance available for *Φ* profile redevelopment. In terms of the RBC fluxes in the Daughter ROIs it should be expected that the Zweifach–Fung effect (higher flux in the higher Q* outlet) will be retained, since the velocities between Daughter 1 and 2 reported in [20] are significantly different: 50% average reduction in Daughter 1 vs. 35% reduction in Daughter 2, compared to the mean velocity in the Parent Branch. No significant effect on RBC flux is expected from the loss of deformability of the cells or other factors, such as RBC aggregation [20,47].

### 3.2. Local Blood Viscosity

**Figure 4:**
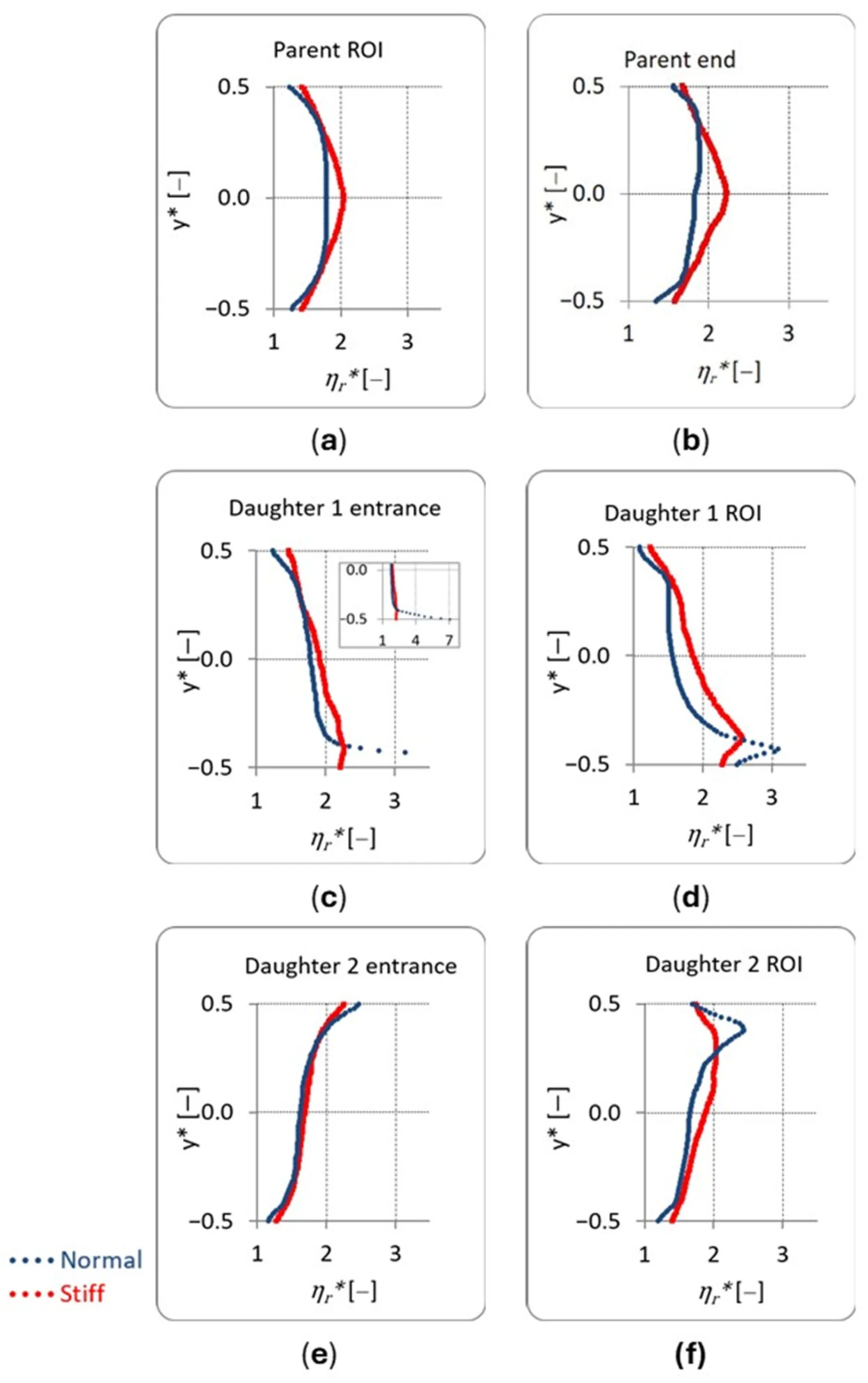
Relative viscosity (η_r_*) profiles against the cross-flow direction y* at different regions and locations in the channel: Parent ROI and Parent-end in panels (**a**,**b**); Daughter 1 branch viscosities in (**c**,**d**); and Daughter 2 branch viscosities in panels (**e**,**f**).

The estimated relative blood viscosity $\eta_{r}^{*}$ profiles for the selected regions and locations in the channel are shown in Figure 4. The profiles, as expected, follow the RBC concentration and deformability dependency as dictated by the apparent viscosity model in Equation (6).

The viscosity profiles of the normal and hardened RBC suspensions shown in Figure 4a,b are broadly symmetric in the Parent branch ROI, although the hardened suspension exhibits a distinctive sharpness and higher viscosity, particularly along the channel centerline. These differences become more pronounced near the Parent-end, where the symmetry of the viscosity profiles is also affected. Downstream of the bifurcation, the highest viscosities occur near the inner walls of the Daughter branches, with the largest values observed at the entrance of the lower-flow branch (Daughter 1). Normal RBC suspensions display more distinct viscosity peaks than the hardened suspensions, with the maxima located slightly away from the inner wall of the Daughter ROIs, mirroring the corresponding hematocrit distributions.

The viscosity profiles in the ROI of the Parent branch imply that the local velocity profile may be affected accordingly. Indeed, in a previous study investigating the effect of RBC rigidity in a straight channel of the same dimensions, it was shown that the stiffer cells result in velocity profiles, which are sharper than these of the normal RBC suspensions [18]. In the same study it was shown that the local hematocrit distribution (i.e., *Φ*(y*)) is significantly sharper than the one for normal RBC suspensions.

Although, intuitively, it would be expected that a sharper radial viscosity profile $\eta_{r}$ would result in a blunter velocity profile, the exact effect of viscosity on the flow is more complex and should be attributed to its shear dependency. For example, a power law viscosity behavior would result in exponential viscosity increase at the center of the flow; therefore, no viscosity bluntness can occur when observing η_r_*. In the case of Carreau-type blood models, which incorporate constant zero-viscosity behavior, a blunter radial viscosity results in sharper velocity profiles. In the present case, the radial viscosity profile is modeled by Equation (6), and its shear dependency should be reclaimed using the local velocity behavior, as in previous works [25].

### 3.3. Apparent Viscosity

**Figure 5:**
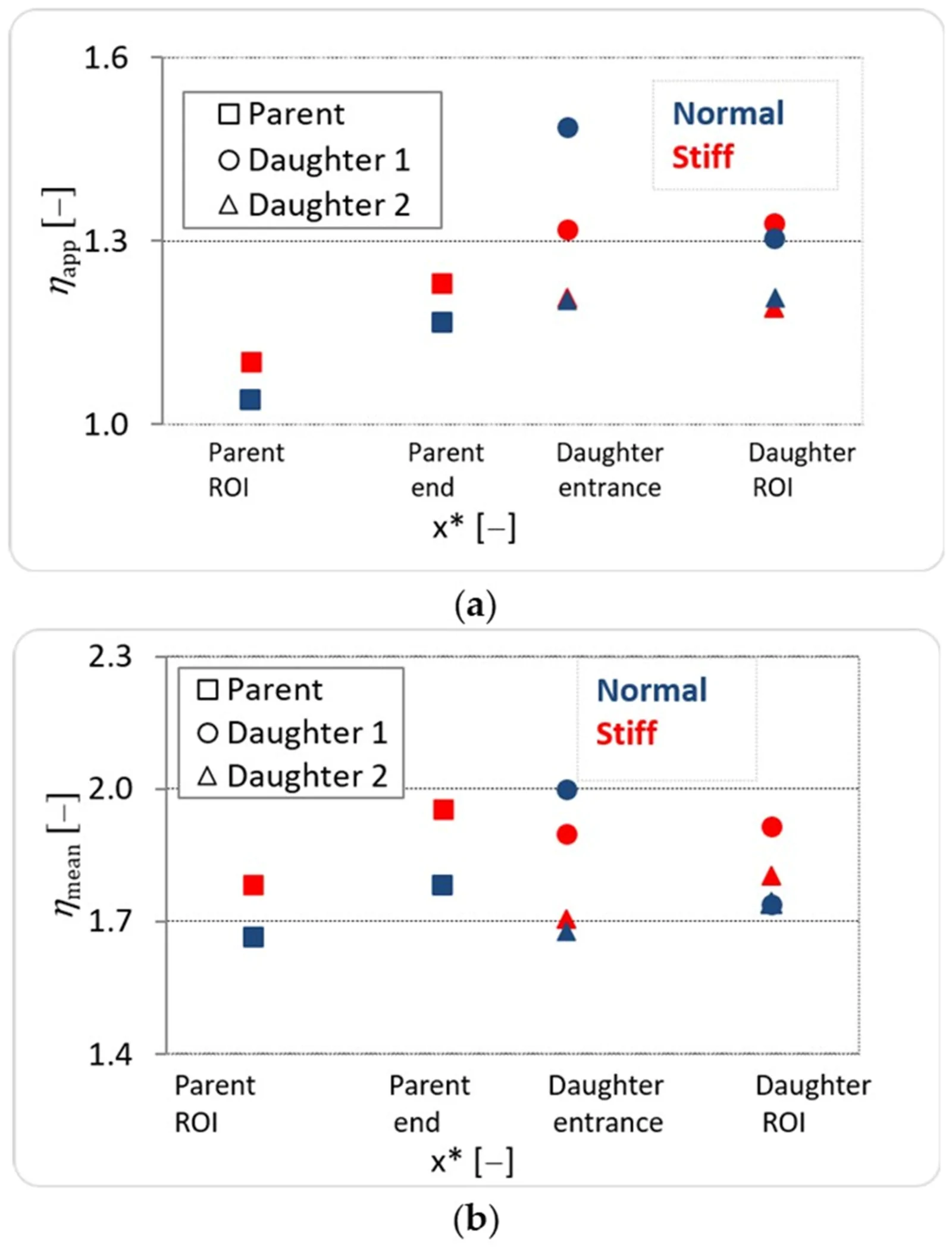
(**a**) Apparent viscosity $\eta_{app}\;$ calculated according to the flow balance approach. (**b**) Mean viscosity values from the viscosity profiles.

The apparent viscosity at the various locations in the channel was calculated as described in Section 2.5 using a flow-balanced approach for λ = 5 and 20 for normal and stiff cells, and the results are shown in Figure 5a. In addition, mean viscosity values were calculated as the arithmetic mean of the viscosity profiles (Figure 5b) to allow us to examine the effect of asymmetry and the extreme values in the viscosity profiles.

The apparent viscosity $\eta_{app}$ of the hardened RBC suspension is consistently higher than that of the normal suspension in the Parent branch. The same applies for the mean viscosity $\eta_{mean}$. As the flow approaches the Y-junction, the apparent viscosity increases for both suspensions, reflecting the enhanced cell accumulation in the bifurcation area. At the entrance of the lower flow rate-ratio daughter branch (Daughter 1), where the local hematocrit is highest for the normal cells, both the apparent and mean viscosities are also greater for the normal RBC suspension. Further downstream at the Daughter ROIs, the apparent viscosity remains elevated compared to the Parent branch; however, it decreases relative to its entrance value. This indicates a partial recovery of the hematocrit flux and flow. For both normal and hardened suspensions, the apparent viscosity is consistently higher in the lower-flow daughter (Daughter 1) and lower in the higher-flow daughter (Daughter 2), as would be expected due to the difference in the shear conditions.

Overall, the viscosity behavior seen in Figure 5a,b indicates that the bulk viscosity of blood increases systematically due to the loss of RBC deformability, except locally in the junction area where the viscosity of the normal samples is higher. It would be expected that the viscosity of the samples would follow the hematocrit recovery, which is expected to take place further downstream the daughter branches as the length of the channel increases.

## 4. Discussion

The present study establishes a direct link between experimentally observed hematocrit distributions and apparent viscosity, through viscosity modeling for normal and stiff RBC suspensions, in specific flow conditions and microchannel geometry. The results highlight that RBC deformability and local concentration, arising from flow distribution and cell partitioning, act synergistically to shape the spatial RBC concentration heterogeneity, which in turn governs the local viscosity characteristics. The apparent viscosity estimated from the local viscosity behavior is considered as an indicator of the potential vascular resistance encountered in a microvascular segment of similar geometry.

### 4.1. Role of Flow and Deformabillity on the Local RBC Concentration

The present results reveal that RBC accumulation develops early, in the Parent-end area (Figure 3), for both the normal and stiffer suspensions. The increase in *Φ_av_* is approximately 10% of the parent branch value. Red blood cell accumulation persists downstream of the junction, at the entrance of Daughter 2, with the stiffer cell suspensions showing a higher clearance compared to the normal RBCs, which show a further increase in *Φ_av_*. The opposite trend is observed at the entrance of Daughter 2, where both stiff and normal cells show a decrease in *Φ_av_*, with the stiffer RBC suspensions showing the larger difference, almost balancing the increase in *Φav* in the Daughter 1 entrance.

The accumulation of RBCs in the vicinity of the bifurcation is consistent with other observations in the literature. RBCs tend to migrate toward the channel center due to shear-induced and lift forces (see parent branch *Φ* in Figure 2a,b), leading to the formation of a cell-rich core and a cell-depleted near-wall layer [13,48]. At bifurcations, this central core can become locally amplified, particularly in regions of flow deceleration as in the proximity of Daughter 1, resulting in a transient increase in local hematocrit. Such behavior has been reported in microvascular networks, where RBCs accumulate near the apex of bifurcations and subsequently redistribute into daughter branches depending on flow partitioning [10]. This redistribution is clearly seen in the *Φ*_av_ values of the Daughter ROIs in Figure 3, where the RBC concentration tends to converge back to the parent branch values. Further, this redistribution is closely linked to the plasma skimming effect, where RBC-rich core regions preferentially feed higher-flow branches, while lower-flow branches receive plasma-enriched fluid [24,49].

The RBC redistribution with preference on the higher flow branch is observed on the *Φ*_av_ values of the normal RBCs in the ROIs of the daughter branches (Figure 3), where the concentration is higher in Daughter 2 (0.5 < Q*_d2_ < 1). Interestingly, the phenomenon is reversed when examining the stiffer RBCs, in which the lower Q* range branch (Daughter 1) receives a higher number of cells compared to Daughter 2. This behavior can be attributed to RBC deformability, which influences the ability of cells to migrate (see the *Φ*_av_ profiles’ differences between the daughter entrances and ROIs in Figure 2) and reorganizes within the flow field, with stiffer cells exhibiting increased lateral migration and enhanced local accumulation [18]. However, when considering the rate at which cells pass through the branches (flux), both normal and stiff samples follow the well-known trend of preferring the higher flow branches [20].

The observed mismatch between mean concentrations when comparing values at parent and daughter branch ROIs might be due to the plasma skimming phenomenon in conjunction with the insufficient length needed to recover the local and bulk concentration characteristics. It has been reported that a length of up to 78 hydraulic diameters is needed to establish an equilibrium state in a rectangular geometry of similar dimensions [50].

As mentioned above, the deformability of the RBCs affects their ability to migrate laterally in the environment of micro-vessel flow. The present results show that the stiffer suspensions show a greater tendency to migrate towards the center of the flow (see the parent branch profiles in Figure 2), and downstream the junction they appear to be more uniformly distributed compared to the normal RBC suspensions. Further, at the center of the channels (y* = 0), the stiff suspensions *Φ*_av_ are either similar or higher than the normal ones. This also agrees with suspension migration theory [28], where particle migration depends on collision frequency and viscosity gradients.

### 4.2. Apparent Viscosity as a Vascular Resistance Indicator

RBC deformability is strongly influenced by cytoplasmic viscosity, which is primarily governed by hemoglobin concentration and state. In healthy RBCs, normal cytoplasmic viscosity (~5 mPa·s) supports high deformability, typically reflected by elongation index (EI) values of ~0.5 [27]. In pathological conditions where cytoplasmic viscosity is increased, deformability is significantly reduced. For instance, in diabetes mellitus, moderate increases in intracellular viscosity and membrane alterations are associated with a ~10–25% decrease in deformability [51]. In RBC dehydration disorders (e.g., hereditary spherocytosis or xerocytosis), elevated mean cell hemoglobin concentration (MCHC) leads to increased cytoplasmic viscosity (up to ~15 mPa·s), resulting in approximately ~40% reduction in EI compared to healthy cells [52]. More pronounced effects are observed in sickle cell disease (SCD), where hemoglobin polymerization under deoxygenated conditions drastically increases effective cytoplasmic viscosity (often exceeding 50 mPa·s), leading to 50–90% reductions in deformability, with some cells exhibiting near-rigid behavior [53,54]. In the present work, where RBC deformability of the stiff cells is reduced to almost half of the normal cells, λ was set to 20.

The results in Figure 4 demonstrate that small changes in concentration, in combination with altered RBC deformability, can lead to amplified viscosity and viscosity asymmetry in the channel branches, especially under uneven flow partitioning. At the Parent-end location (Figure 4b) the viscosity of the stiffer suspension has already been elevated at the channel center. Viscosity asymmetry is clearly apparent when examining the viscosity profiles downstream of the Parent-ROI area. At the entrance of the daughter branches the viscosity reaches a maximum near the inner walls, with the effect more pronounced for normal suspensions, reflecting the impact of RBC concentration, which also exhibits the highest values therein (see Figure 2c). Further downstream at the channel ROIs, the viscosity peak remains more pronounced for the normal RBC suspensions; however, its value has been largely reduced. In the same region, the viscosity of the stiff suspensions appears to reach higher values compared to that of normal cells away from the inner walls of the branches.

In order to translate the relative viscosity profiles of Figure 4 into apparent viscosities in each location, and consequently, to reflect the vascular resistance in the area, a flow rate balance approach was adopted (Equation (8)), according to the work of Damiano et al. [43] and Macoscko [44]. From a fluid mechanics perspective, this approach is considered as more appropriate since it is governed by flow-weighted contributions (flow rate balance). Figure 5 illustrates the apparent viscosity *η*_app_ and *η*_mean_ at the various locations in the channel. Notably, the viscosity of the stiff suspensions is higher in the parent channel and daughter ROIs. At the entrance of Daughter 1, the apparent viscosity of the normal samples is significantly higher, while at the ROI of the same daughter, the viscosities of both suspensions converges to similar values. Apparently, the estimation of *η*_mean_ is influenced more by the peaks of the relative viscosity near the inner walls of the daughter branches, rather than differences seen at the central regions of the channel (see the distribution of *η*_app_ in Figure 4d,f). This implies that small regions of high cell concentration in the microvessel could have a profound effect in the bulk resistance characteristics in the area.

In pathological conditions, deformability changes may alter local RBC perfusion and distribution, and consequently, vascular resistance in the microcirculatory system. For example, microvascular resistance is significantly altered in diabetes mellitus, largely due to changes in RBC deformability, increased blood viscosity, and endothelial dysfunction; hyperglycemia induces non-enzymatic glycation of hemoglobin and membrane proteins, leading to increased cytoplasmic viscosity and reduced RBC deformability [51]. Experimental and clinical studies have demonstrated that these rheological alterations result in reduced capillary perfusion, increased flow heterogeneity, and impaired oxygen delivery, particularly in tissues with high metabolic demand [55].

**Figure 6:**
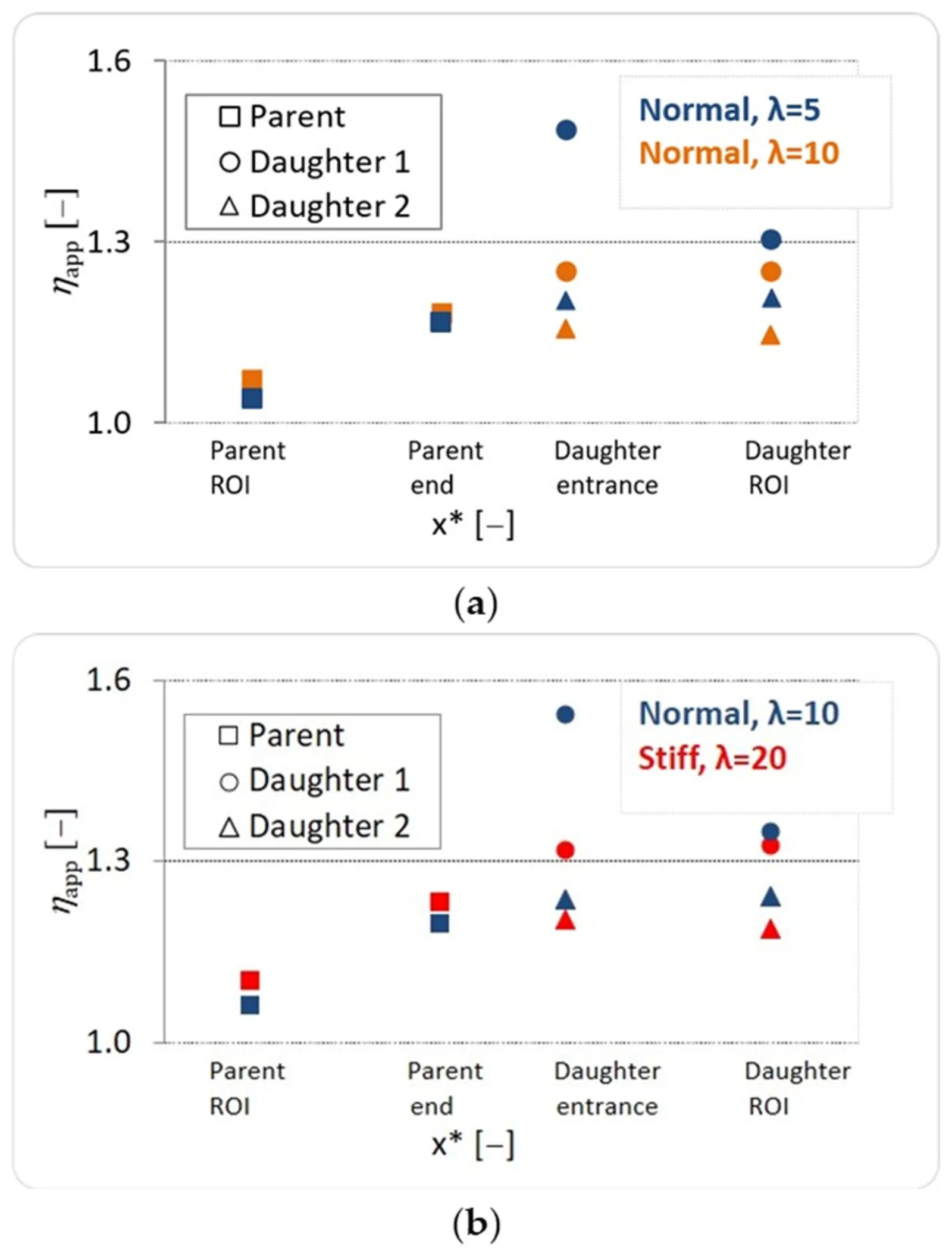
(**a**) Apparent viscosity *η*_app_ between normal cases having different λ values of 5 and 10, and (**b**) apparent viscosity values with λ = 10 for the normal RBC case and 20 for the stiffer RBC case.

To explore the impact of the recently reported cytosol viscosity contrast value of λ = 10 in [39], a comparison between the apparent viscosities of the normal case for λ = 5 and 10, with $\Phi_{m}$ = 0.95 for both, was attempted and presented in Figure 6.

It is evident in Figure 6a that, although in the parent branch the discrepancy between the cases is very small (well below 1%), in the daughter branches the differences in *η*_app_ increase. At the entrance of the lower flow rate branch of Daughter 1, an increase of approximately 15% is calculated for the viscosity of the normal cells, following the mean concentration pattern *Φ*_av_ seen in Figure 3. Comparing *η*_app_ of the normal against the stiffer RBC cases in Figure 6b, using values of λ = 10 and 20 and Φ_m_ = 0.95 and 0.70 for the normal and stiffer cases respectively, it is apparent that the general behavior in the channel remains similar to that in Figure 5a. However, the viscosity of the normal samples seems to be higher than that of the stiffer cases within all ROIs in the daughter branches.

It should be noted, however, that the comparison made in Figure 6b lacks a correction of the λ value for the stiffer RBC cases, as the experimental value of 20 utilized here is not extracted by the methodology used in [39], which requires further investigation. Nevertheless, the comparisons illustrate the need for more accurate probing into the physical aspects of RBC properties, as model outputs can exhibit non-linear sensitivity to specific parameters [56,57].

Overall, the apparent viscosity estimates obtained in this study provide a direct rheological measure that can be related to the hydraulic resistance of microvascular segments. Since vascular resistance depends on fluid viscosity for a given vessel geometry and flow regime, the observed local increases in apparent viscosity indicate corresponding increases in the pressure drop required to sustain blood flow through bifurcating microvessels. The results demonstrate that these resistance changes arise not only from the intrinsic rheological properties of stiffened RBC suspensions, but also from deformability-dependent redistribution of cells and the resulting local hematocrit heterogeneity at bifurcations. Consequently, pathological reductions in RBC deformability may modify microvascular resistance indirectly by altering RBC partitioning and concentration, in addition to directly increasing suspension viscosity. These findings highlight the importance of incorporating spatially varying hematocrit and deformability into models of the microcirculation; they also provide a framework for estimating local vascular resistance from experimentally measured RBC distributions in physiologically relevant microvascular geometries.

## 5. Conclusions

A key contribution of this work is demonstrating that RBC deformability modifies viscosity not only directly, but also indirectly via a redistribution of the RBCs in the daughter branches. Accurate modeling of microvascular flow requires incorporation of both deformability-dependent viscosity changes and altered hematocrit distribution. The findings suggest that resistance in bifurcating networks cannot be predicted solely from bulk hematocrit but requires local RBC distribution-informed modeling. Future work shall extend the present methodology to pathological blood samples, integrating experimentally measured red blood cell deformability with spatial hematocrit distributions and modeling to establish clinically relevant relationships between cellular mechanics and microvascular flow resistance.

## Data Availability

The data that support the findings of this study are available from the corresponding author upon reasonable request.
